# Impact of lifestyle behaviors on clinical outcomes of steps 1 and 2 of periodontal therapy in stage iii-iv periodontitis: a cohort study

**DOI:** 10.1007/s00784-025-06668-9

**Published:** 2025-11-24

**Authors:** Federica Romano, Giacomo Baima, Melanie Llerena-Velasquez, Lisa Siccardi, Giacomo Tonello, Marta Vernero, Davide Giuseppe Ribaldone, Mario Aimetti

**Affiliations:** 1 Department of Surgical Sciences, C.I.R. Dental School, University of Turin, Turin, Italy; 2 Department of Medical Sciences, Gastroenterological Clinic, University of Turin, Turin, Italy; 3 Department of Surgical Sciences, C.I.R. Dental School, University of Turin, Via Nizza 230, Turin, Turin, 10126 Italy

**Keywords:** Health behavior, Lifestyle, Longitudinal studies, Oral health, Periodontal diseases

## Abstract

**Objective:**

Given the inconsistent evidence in the literature, the aim of this prospective cohort study was to evaluate the impact of lifestyle on the clinical response to Steps 1 and 2 of periodontal therapy in patients with severe periodontitis.

**Materials and methods:**

Patients with untreated stage III/IV periodontitis received non-surgical periodontal therapy and were re-evaluated 3 months later (T1). Based on baseline self-administered questionnaires, four lifestyle factors - physical activity, dietary habits, smoking, and alcohol consumption- were scored as either healthy (1 point) or unhealthy (0 point). An overall healthy lifestyle variable was calculated for each patient by summing the individual scores (ranging from 0 to 4 points), and its impact on clinical outcomes was analyzed. The primary outcome of the study was disease control (≤ 4 residual probing pocket depth (PPD) ≥ 5 mm).

**Results:**

A total of 120 patients were treated. At T1, a statistically significant association was observed between the overall lifestyle score and treatment response (*p* = 0.029). Specifically, 55.1% of patients with healthy lifestyle behaviors (score 3–4) achieved the primary endpoint, compared to 29.0% and 32.5% of those with partial (score 2) or poor health attentiveness (score 0–1), respectively. Additionally, health-conscious individuals demonstrated better control of gingival inflammation, despite having comparable oral hygiene levels (*p* < 0.001). In the logistic regression model, attentiveness to one’s health (OR 3.39, 95% CI 1.13–9.47, *p* = 0.020), normal body weight (OR 2.93, 95% CI 1.12–7.70, *p* = 0.029) and percentage of sites with PPD ≥ 6 mm at baseline (OR 0.90, 95% CI = 0.84–0.97, *p* = 0.006) were significantly associated with treatment outcome.

**Conclusion:**

Adherence to positive healthy lifestyle factors contributes to better clinical outcomes following periodontal therapy in patients with stage III/IV periodontitis.

**Clinical relevance:**

The adoption of healthy lifestyle behaviors should be considered as part of the overall management strategy for patients with severe periodontitis.

**Supplementary Information:**

The online version contains supplementary material available at 10.1007/s00784-025-06668-9.

## Introduction

Periodontitis is a chronic inflammatory disease that causes progressive and irreversible destruction of the tooth-supporting structures. Its global prevalence exceeds 50% of the population, with severe forms affecting almost 15% of individuals [1–4]. Severe periodontitis is currently the sixth most prevalent non-communicable disease (NCD) worldwide and is the leading cause of tooth loss in adults, with a detrimental effect on masticatory function, self-esteem, and overall well-being [5–7]. The etiopathogenesis is complex, but it is primarily characterized by an excessive inflammatory host response to microbial dysbiosis, modified by genetic, epigenetic and environmental factors [3, 8–10]. Thus, the effective and sustained dental biofilm control and the management of risk factors are considered primary components of periodontitis prevention and therapy [11].

Recently, unhealthy diet, physical inactivity, cigarette smoking and alcohol use have been identified by the World Health Organization (WHO) as the four modifiable behavioral risk factors for NCD prevention [12]. These unhealthy behaviors increase the level of circulatory pro-inflammatory mediators leading to low-grade systemic inflammation (LGSI), but also cause overproduction of reactive oxygen species (ROS) resulting in oxidative stress [13–15]. While the benefits of lifestyle have been widely promoted in public health policy and extensively studied for other NCDs [16–19], their impact on periodontal health has only recently begun to receive attention. In addition to the well-established links between cigarette smoking and alcohol abuse with periodontitis prevalence and response to treatment, the influence of diet and physical activity are still emerging [20–25].

Low levels of physical activity and unhealthy diet have been significantly associated with higher gingival inflammation and increased number of sites with deep probing depth [26] and severe clinical attachment loss [27]. Their combination was also found to increase the odds of severe periodontitis by 10 times [23]. In a recent prospective cohort study, a poor lifestyle – characterized by poor sleep quality, smoking, drinking, low adherence to the Mediterranean diet, physical inactivity and high stress - was negatively associated with the clinical response to Steps 1 and 2 of periodontal therapy [28]. Specifically, these unhealthy behaviors were linked to a higher number of deep residual pockets and a lower likelihood of achieving treatment goals. However, the majority of patients in the study had stage II periodontitis, leaving the influence of negative lifestyle factors on patients with more severe forms of the disease (stages III and IV) unclear. Additionally, while previous studies have mostly examined the impact of individual lifestyle factors on periodontal health, the combined effect of these factors still requires further scientific investigation. The rationale for combining these lifestyle factors into a composite score aligns with the WHO’s ‘health behavior clusters’ concept, which highlights the cumulative and interactive effects of multiple health behaviors in NDS prevention [18].

Given the limited data currently available, this study aimed to assess the impact of lifestyle—measured by the combination of diet, physical activity, smoking, and alcohol use—on the clinical response to Steps 1 and 2 of periodontal therapy in patients diagnosed with severe periodontitis.

## Methods

### Study design and setting

The present prospective monocentric cohort study was carried out at the Section of Periodontology, C.I.R. Dental School, University of Turin (Italy) from March 2021 to February 2024. All procedures were conducted in accordance to the Good Clinical Practice Guidelines and the principles of the Declaration of Helsinki. Ethical approval (Ethical Committee of the “AOU Città della Salute e della Scienza” of Turin, protocol number 00066/2021) and informed written consent from participants were obtained prior to enrollment. The STROBE statement (Strengthening the Reporting of Observational Studies in Epidemiology) for observational studies with a cohort design was applied for reporting [29].

## Sample characteristics and recruitment

Participants were consecutively recruited from those attending consultation visits, based on the following inclusion criteria: age >18 years; diagnosis of stage III generalized or stage IV periodontitis (any grade) [30]; presence of at least 20 teeth, with at least 10 sites showing probing depth (PPD) ≥ 6 mm and radiographic alveolar bone loss; ability to communicate in Italian to respond to the questionnaires; and willingness to provide informed consent.

The presence of any of the following criteria led to exclusion from the study: pregnancy or breastfeeding; periodontal therapy in the past 12 months; presence of systemic diseases that modulate the clinical response to periodontal treatment (e.g., uncompensated diabetes mellitus); use of antibiotics, steroidal and/or non-steroidal anti-inflammatory drugs in the three weeks prior to the visit, and patients on medications that can induce gingival enlargement (immunosuppressants, estrogen-progestins, calcium antagonists, anticonvulsants). While cigarette smokers were included, individual using other forms of tobacco or nicotine, such as vaping, waterpipe, or smokeless tobacco, were excluded to maintain a consistent definition of smoking exposure.

## Lifestyle behavior and demographic measurements

During the first visit, one week prior to starting periodontal therapy (baseline, T0), data on sociodemographic characteristics (age, gender, education level, body mass index [BMI]), medical and dental history, and lifestyle habits were collected for each participant. In the case of diabetes mellitus, only patients with adequate metabolic control were enrolled, as determined by hemoglobin A1c (HbA1c) levels ≥ 7% [31].

Patients completed two questionnaires on physical activity and dietary habits, administered by two operators who were not involved in the clinical phase of the study. These operators carefully verified the completeness of the questionnaires at the time of collection. To assess physical activity, the validated Italian translation of the short form of the International Physical Activity Questionnaire (IPAQ) was used. It measures the frequency and average duration of vigorous and moderate recreational and occupational physical activities, walking (regardless of whether during work or leisure), as well as sedentary activities over the past 7 days [32]. Total physical activity is summarized as a continuous variable, calculated in metabolic equivalent (MET)-minutes per week or categorized in levels. MET is estimated by multiplying the time spent on each activity per week by the MET intensity of that activity. According to the IPAQ, weekly physical activity is classified into three levels: good (at least one hour of moderate physical activity performed every day), moderate (at least half an hour of moderate activity performed almost every day) or poor (below the moderate level or absent).

The second questionnaire on dietary habits was a simplified version of the alternate Mediterranean Diet (aMED) scoring system [33]. Each participant was asked to specify their diet type (Mediterranean, vegetarian, vegan) and the weekly frequency of intake (never, less than once a week, once a week, twice a week, at least three times a week, every day) for various food macro-categories (carbohydrates, legumes, proteins, nuts, fresh fruits, vegetables, dairy, yogurt, eggs, non-carbonated beverages, carbonated beverages, alcoholic beverages). Foods consumed at least three times a week or daily were considered “prevalent” in the subject’s diet. Conversely, foods that the participant never consumed or only rarely were considered “excluded” from the diet. Additionally, any food allergies and intolerances were recorded. Based on the type and frequency of food consumption, participants were classified as having a varied and balanced diet or not [34]. For each food group, a score of 1 was assigned if it was consumed almost daily or 0 if it was not consumed daily (except for carbonated beverage for which the score was reversed), excluding alcohol due to separate categorization. The total dietary score, calculated by summing the individual scores for each food group, ranged from 0 to 9, with higher scores indicating greater dietary variety. Smoking status (current smoker, former smoker, never smoker), the number of cigarettes smoked daily, smoking duration, were self-reported by participants in an interview before the initiation of the periodontal therapy. Participants were classified as former smokers if they quit the habit prior to the interview. Additionally, the frequency of alcohol consumption was self-reported and categorized as follows: never (never consumed alcohol or not in the past 12 months), infrequent (less than once a week), 1–2 times per week, 3–4 times per week, and daily or almost daily. Smoking status (current smoker, former smoker, never smoker), the number of cigarettes smoked daily, smoking duration, were self-reported by participants in an interview at the completion of the periodontal therapy. Participants were classified as *former smokers* if they quit the habit prior to the interview. Additionally, the frequency of alcohol consumption (never, occasional, at least twice a week, almost every day) was also self-reported during the same interview.

A composite exposure variable, referred to as the overall healthy lifestyle score, was created to assess the lifestyle patterns of the enrolled participants (Table 1). One point was assigned for each of the following criteria: non-smoking status (including former smokers), intense weekly physical activity, adherence to a balanced diet (defined as a dietary score above the population median of 5 [24]), and alcohol consumption limited to occasional use or none at all [20]. Participants who did not meet these criteria received a score of 0 for the respective component. The overall score, was calculated by summing the points across the four lifestyle factors, yielding a final score ranging from 0 (unhealthy lifestyle) to 4 (healthy lifestyle). To ensure a balanced distribution of participants across groups, the overall scores were categorized into three groups: 0–1, 2, and 3–4. Participants with scores of 0 or 1 were combined into one group, and those with scores of 3 or 4 into another. These cut-off points were also informed by previous studies using similar composite lifestyle scores, in which lifestyle behaviors were categorized into low, moderate, and high adherence groups, reflecting their clinical relevance to health outcomes [18, 20].

**Table 1:** Overall healthy lifestyle variable

Lifestyle component	Definition	Score
Smoking status	Non-smoker/Former smoker	1
Physical activity	Intense weekly physical activity	1
Diet	Adherence to a balanced diet (aMED score > 5)	1
Alcohol consumption	Rarely or never consumed alcohol	1
Total score range	Sum of all components	0–4

aMED, alternate Mediterranean Diet scoring system.

## Periodontal examination

A complete periodontal examination was performed at T0 and three months after the completion of Steps 1 and 2 of periodontal therapy (T1) by two calibrated examiners. They were different from the interviewers and were unaware of the participants’ lifestyles. Intra- and inter-examiner reproducibility for probing depth (PPD) and gingival recession (REC) were assessed twice by repeated measurements in 10 patients not included in the study using the intra-class correlation coefficient (ICC). ICC values for intra-examiner and inter-examiner reproducibility ranged from 0.89 to 0.96 and 0.88 to 0.94, respectively.

The following clinical parameters were recorded at six sites for each tooth using a graduated periodontal probe (PCP 15, Hu-Friedy, Chicago, IL, USA): presence/absence of plaque (PI); presence/absence of bleeding on probing (BoP); PPD measured as the distance in mm from the free gingival margin (GM) to the base of the pocket/sulcus; REC measured as the distance in mm between GM and the cementoenamel junction (CEJ); CAL measured in mm from CEJ to the most apical point of the pocket/sulcus [35]. The degree of tooth mobility was also recorded [28]. The percentage of sites displaying PI or BoP out of the total number of sites examined in the full mouth was calculated and expressed as full mouth plaque score (FMPS) and full mouth bleeding score (FMBS), respectively.

## Periodontal therapy

All participants underwent Steps 1 and 2 of periodontal therapy [11, 36, 37]. In step 1, patients were instructed and motivated regarding proper home hygiene practices using an electric toothbrush and interdental devices suitable for the anatomy of the interproximal spaces. Professional removal of supragingival biofilm and calculus deposits was also performed using ultrasonic instruments (Cavitron Select SPS, Dentsply Sirona, Rome, Italy). This phase lasted 1–2 weeks, depending on the individual compliance and response. If patients did not achieve a good biofilm control at home, which in return did not reduce the superficial inflammation, additional sessions of oral hygiene instructions and motivation were carried out.

During step 2, a Conventional Stage Debridement was carried out in quadrants over four weekly appointments. Subgingival instrumentation was performed using manual instruments (Gracey curettes, Hu-Friedy, Chicago, IL) and ultrasonic tools, without the additional use of antiseptic agents or antibiotic therapy, under local anesthesia. At each session, patients were instructed and motivated regarding proper oral hygiene practices. One month after completing non-surgical therapy, all patients were recalled for a re-evaluation of home biofilm control and motivational reinforcement. At the end of the study, smokers received counseling to quit or at least reduce their daily cigarette consumption [9]. Additionally, all patients were informed about how a healthy lifestyle (regular physical activity, adequate diet, abstinence from smoking and alcohol) is crucial for the prevention and treatment of numerous chronic conditions, such as periodontitis, and that achieving a state of health requires a commitment to modify inadequate lifestyle habits. Patients who did not achieve the predefined endpoints were advised to proceed to Step 3 therapy (re-instrumentation or surgical treatment as indicated).

### Study outcomes

The primary outcome variable was disease control assessed at the patient level, defined as achieving no more than four residual periodontal pockets with PPD ≥ 5 mm at the completion of Steps 1 and 2 of periodontal therapy [38]. The mean change in other clinical periodontal variables during the 3-month observation period was considered a secondary outcome of the study.

## Sample size calculation and data analysis

The sample size was calculated based on the 49% of patients achieving the treatment endpoint, as reported by Feres et al. [38]. This calculation assumed a 50% reduction in success due to unhealthy lifestyle factors, with two-thirds of subjects with severe periodontitis exhibiting unhealthy behaviors [23]. Given a power of 80% and an alpha level of 5%, the required sample size was determined to be 120 individuals.

All statistical analyses were performed using the SPSS statistical software package (version 28, IBM), with the significance level set at 5%. Summary statistics included means (standard deviations) or median (interquartile range) for continuous quantitative variables and frequency distributions for categorical variables. Normality of the quantitative variables was assessed using the Shapiro-Wilk test. Within-group changes in quantitative variables were analyzed using the paired t-test (for FMBS, PPD, CAL) or the Wilcoxon test (for FMPS, REC, number of teeth, percentage of sites with PPD 4–5 mm and BoP, and percentage of sites with PPD ≥ 6 mm), as appropriate, based on data distribution. Differences in clinical parameters among the three groups, stratified according to the overall healthy lifestyle score, were assessed using one-way ANOVA or the Kruskal-Wallis test, followed by Tukey test or Dunn’s test with a correction for multiple comparisons. Qualitative variables were analyzed using the Chi-square test.

To evaluate the effect of baseline periodontitis severity, the overall healthy lifestyle score, and BMI on the primary treatment outcome, a multiple logistic regression model was constructed. BMI was categorized as a binary variable: normal weight (18.5–25 kg/m²) versus overweight/obese (> 25 kg/m²). The model included adjustments for potential confounders, specifically age and full-mouth plaque score (FMPS) at 3 months post-therapy. To account for potential confounding factors not included in the primary model, a sensitivity analysis was performed. In this secondary model, gender and hypertension were added as covariates to the multivariable logistic regression to evaluate the robustness of the observed associations. Other systemic diseases were not included due to the low prevalence in the sample. Odds ratios (ORs) and 95% confidence intervals (CIs) were calculated.

## RESULTS

### Participants characteristics

The characteristics of the sample are summarized in Table 2. A total of 120 patients (mean age of 54.6 ± 11.7 years) were consecutively included in the study, with 63.3% having stage III periodontitis and 36.7% having stage IV periodontitis. Of the participants, 64 were women (53.3%), and the majority were of Caucasian ethnicity (85.8%).

Regarding lifestyle habits, 20% of the sample reported being smokers, with an average of 10.5 ± 6.3 cigarettes smoked per day (range: 2–30). A minority (5%) were former smokers who had quit an average of 9.0 ± 1.2 years ago (range: 8–10 years). Fifty-nine participants (49.2%) followed a varied and balanced diet, and 76 (63.6%) reported not consuming alcoholic beverages or consuming them rarely. According to the IPAQ questionnaire on weekly physical activity, 45% of the sample (*n* = 54) were categorized as “very active,” 25% (*n* = 30) as “moderately active,” and the remaining 30% (*n* = 36) were classified as “sedentary”. The average MET value was 5426.9 ± 7854.3 min per week, and the average BMI was 24.0 ± 3.4 kg/m², with approximately one-third of participants being overweight or obese (32.5%).

**Table 2:** Demographic, medical and behavioral characteristics of the overall sample

Variables	Total (*N* = 120)
Age (years), mean (± SD) Gender, female, n (%)	54.6 (± 11.7) 64 (53.3)
Ethnicity, n (%)	
Caucasian	103 (85.8)
Afro-American	6 (5.0)
Asian	2 (1.7)
Hispanic	7 (5.8)
Other	2 (1.7)
Periodontitis Stage, n (%)	
III	76 (63.3)
IV	44 (36.7)
Systemic diseases, n (%)	
Controlled Type 2 diabetes	4 (3.3)
Osteoporosis	1 (0.8)
Hypertension	20 (16.7)
Dyslipidaemia	2 (1.7)
Respiratory diseases	3 (2.5)
Heart diseases	5 (4.2)
Gastrointestinal diseases	8 (6.7)
Medication intake, yes, n (%)	39 (32.5)
Smoking, n (%)	
No	90 (75.0)
Current smoker	24 (20.0)
Former smoker	6 (5.0)
Mediterranean diet adherence, n (%)	59 (49.2)
Physical activity, n (%)	
Inactive	36 (30.0)
Moderately active	30 (25.0)
Active	54 (45.0)
MET-min/week, mean (± SD)	5426.9 (± 7854.3)
Alcohol intake, n (%)	
Never	51 (42.5)
< 1 day a week	25 (20.8)
1 day a week	12 (10)
3 days a week	9 (7.5)
Every day	23 (19.2)
BMI (kg/m^2^), mean (± SD)	24.0 (± 3.4)

MET-min/week, metabolic equivalent-minutes/week; BMI, Body mass index.

Participants were stratified based on their healthy lifestyle score into three groups: 40 (17 men and 23 women, mean age 54.1 ± 11.8 years) in the unhealthy (score 0–1), 31 (15 men and 16 women, mean age 56.9 ± 9.9 years) in the intermediate (score 2), and 49 (24 men and 25 women, mean age 53.7 ± 12.8 years) in the healthy (score 3–4) lifestyle groups.

### Clinical outcomes after steps 1 and 2 of periodontal therapy

All patients completed Steps 1 and 2 of periodontal therapy and were re-evaluated at T1. No patient experienced adverse events or required antibiotic therapy during the study. A total of 49 subjects (40.8%) achieved the primary treatment endpoint, showing a statistically significant reduction in all clinical parameters compared to baseline values (all *p* ≤ 0.01), as detailed in Supplementary Table 1. When participants were stratified according to their healthy lifestyle score, the three groups were comparable across all periodontal parameters assessed at T0, as shown in Table 3.

**Table 3:** Periodontal clinical variables at baseline according to the lifestyle groups

Variables	Lifestyle groups							
	Unhealthy (score 0–1) (*n* = 40)		Moderately healthy (score 2) (*n* = 31)		Healthy (score 3–4) (*n* = 49)		P*	
	Mean (SD)	Median (IQR)	Mean (SD)	Median (IQR)	Mean (SD)	Median (IQR)		
N° teeth	26.0 (4.3)	27.0 (3.0)	25.5 (3.9)	26.0 (4.0)	26.3 (3.5)	27.0 (4.5)	0.602	
FMPS (%)	76.0 (18.3)	75.0 (28.5)	79.2 (14.9)	79.0 (18.0)	72.7 (17.3)	76.0 (16.5)	0.270	
FMBS (%)	72.3 (23.4)	75.0 (37.8)	69.2 (20.2)	74.0 (30.0)	76.6 (16.8)	78.0 (23.0)	0.321	
PPD (mm)	4.4 (0.6)	4.4 (0.9)	4.2 (0.7)	4.2 (1.1)	4.3 (0.7)	4.2 (0.9)	0.644	
% PPD 4–5 mm	26.3 (12.1)	24.6 (21.1)	24.0 (9.9)	22.6 (18.1)	25.0 (11.7)	23.2 (18.8)	0.851	
% PPD ≥ 6 mm	14.9 (8.3)	13.9 (10.5)	13.6 (5.9)	13.5 (8.8)	12.1 (5.5)	11.3 (7.4)	0.324	
REC (mm)	0.6 (0.5)	0.5 (0.9)	0.8 (0.7)	0.6 (1.2)	0.6 (0.6)	0.5 (0.8)	0.847	
CAL (mm)	5.0 (0.9)	5.0 (1.2)	5.0 (1.1)	4.9 (1.4)	4.9 (1.0)	4.8 (1.4)	0.985	

SD, Standard deviation; IQR, Interquartile Range; FMPS, Full Mouth Plaque Score; FMBS, Full Mouth Bleeding Score; PPD, Probing Pocket Depth; %PPD 4–5 mm, percentage of sites with probing depth of 4–5 mm and bleeding; % PPD ≥ 6 mm, percentage of sites with probing depth ≥ 6 mm; REC, Gingival Recession; CAL: Clinical attachment level; *P value for comparisons among groups.

Non-surgical periodontal therapy resulted in a significant reduction in all periodontal parameters across all lifestyle groups when compared to baseline values (all *p* < 0.001). However, lifestyle was found to have a statistically significant impact on treatment response (*p* = 0.029). Specifically, 55.1% of patients who adhered to a healthy lifestyle achieved the therapy outcome, compared to 29.0% and 32.5% of those who were partially or minimally attentive to their health, respectively. As summarized in Table 4, individuals with healthier lifestyles demonstrated more favorable clinical results three months post-therapy. They exhibited lower FMBS values (13.6 ± 5.5%) than those with unhealthy lifestyle behaviors (18.5 ± 8.2%, *p* = 0.005), a smaller percentage of sites with PPD of 4–5 mm and persistent inflammation (8.1 ± 6.9% vs. 15.1 ± 5.9%, *p* < 0.001), and fewer residual pockets with PPD ≥ 6 mm (4.2 ± 3.7% vs. 7.1 ± 3.8%, *p* = 0.001), although mean FMPS values were comparable between the groups. Consistent with these findings, the healthy lifestyle group also demonstrated lower mean PPD and mean CAL values (*p* < 0.001 and *p* = 0.031, respectively). In contrast, no significant differences in periodontal parameters were observed between patients who were partially or minimally attentive to their health.

**Table 4:** Periodontal clinical variables 3 months after steps 1 and 2 of treatment according to the lifestyle groups

Variables	Lifestyle groups													
	Unhealthy (score 0–1) (*n* = 40)				Moderately healthy (score 2) (*n* = 31)			Healthy (score 3–4) (*n* = 49)			P*			
	Mean (SD)	Median (IQR)		Mean (SD)			Median (IQR)	Mean (SD)		Median (IQR)				
N° teeth	24.3 (5.1)		25.5 (5.0)	24.4 (4.3)		25.0 (4.0)		25.6 (3.9)	26.0 (5.0)			0.555		
FMPS (%)	15.6 (8.6)		13.5 (9.0)	15.0 (5.8)		14.0 (9.0)		13.1 (5.1)	13.0 (7.0)			0.447		
FMBS (%)	18.5 (8.2)		17.5 (11.3)	15.3 (7.3)		13.0 (11.0)		13.6 (5.5)	13.0 (6.0)			0.005^B^		
PPD (mm)	3.3 (0.7)		3.3 (1.0)	3.1 (0.6)		3.1 (0.8)		2.7 (0.5)	2.7 (0.5)			0.001 ^A^		
% % PPD 4–5 mm	15.1 (5.9)		14.6 (10.1)	12.1 (7.4)		11.6 (8.2)		8.1 (6.9)	6.2 (7.8)			< 0.001 ^A^		
% PPD ≥ 6 mm	7.1 (3.8)		6.9 (6.3)	6.3 (4.1)		5.9 (8.8)		4.2 (3.7)	3.1 (4.8)			0.002 ^B^		
REC (mm)	0.8 (0.6)		0.7 (0.8)	0.8 (0.8)		0.9 (1.1)		0.7 (0.7)	0.6 (0.7)			0.816		
CAL (mm)	4.1 (1.0)		4.2 (1.4)	3.9 (1.2)		4.0 (1.4)		3.4 (0.9)	3.3 (1.0)			0.024 ^C^		

SD, Standard deviation; IQR, Interquartile Range; FMPS, Full Mouth Plaque Score; FMBS, Full Mouth Bleeding Score; PPD, Probing Pocket Depth; %PPD 4–5 mm, percentage of sites with probing depth of 4–5 mm and bleeding; % PPD ≥ 6 mm, percentage of sites with probing depth ≥ 6 mm; REC, Gingival Recession; CAL: Clinical attachment level; *P value for comparisons among groups; Superscripts indicate statistically significant differences between healthy and unhealthy groups: ^A^ < 0.001; ^B^ < 0.01; ^C^ < 0.05.

To account for potential baseline differences, the mean change in clinical parameters from baseline to 3-month follow-up was also analyzed (Supplementary Table 2). The healthy lifestyle group exhibited a significantly greater mean reduction in FMBS and PPD, along with a higher CAL gain, compared to the unhealthy group.

Finally, Table 5 presents factors associated with the success of non-surgical periodontal therapy in a multiple logistic regression model, adjusted for age and home biofilm control at T1. The severity of periodontitis, measured by the percentage of sites with PPD ≥ 6 mm at T0, reduced the probability of achieving the treatment outcome (OR 0.90, 95% CI = 0.84–0.97, *p* = 0.006). In contrast, being attentive to one’s health (OR = 3.39, 95% CI = 1.13–9.47, *p* = 0.020) and having normal weight (OR = 2.93, 95% CI = 1.12–7.70, *p* = 0.029) significantly increased the probability of achieving the treatment outcome. The inclusion of gender and hypertension did not change the strength or direction of the associations between lifestyle factors and periodontal outcomes (Supplementary Table 3).

**Table 5:** Multiple logistic regression model with predictors of primary treatment outcome (a maximum of 4 sites with PPD ≥ 5 mm at T1)

Variables	OR	CI 95%	*P*-value
Age (years)	0.98	0.95–1.02	0.346
FMPS at T1 (%)	1.01	0.95–1.07	0.739
% PPD ≥ 6 mm at T0	0.90	0.84–0.97	0.006
Lifestyle patterns			
Unhealthy	Ref.	-	-
Moderately healthy	1.21	0.39–3.80	0.738
Healthy	3.39	1.21–9.46	0.020
BMI (kg/m^2^)			
Overweight	Ref.	-	-
Normal weight	2.93	1.12–7.69	0.029
Intercept	2.08	-	0.521

PPD, Periodontal pocket depth; FMPS, Full Mouth Plaque Score; T0, baseline; T1, 3 months after Steps 1 and 2 of periodontal therapy; % PPD ≥ 6 mm, percentage of sites with probing depth ≥ 6 mm; BMI, Body mass index; OR, Odds ratio; 95% IC, 95% Interval confidence.

## DISCUSSION

This longitudinal cohort study demonstrates a statistically and clinically significant association between lifestyle-related behaviors and the clinical outcomes of periodontal therapy in patients with severe periodontitis (stage III and IV). Three months after completing steps 1 and 2 of periodontal therapy, only 32.5% of patients who did not follow a balanced diet, led a sedentary lifestyle, habitually consumed alcoholic beverages and were smokers, reached the treatment endpoint (≤ 4 sites with PPD ≥ 5 mm). In contrast, 55.1% of patients with healthy lifestyle behaviors achieved the same outcome. Furthermore, individuals who were mindful of their lifestyle exhibited a greater improvement in inflammatory parameters and a more significant reduction in PPD values. The percentage of deep residual pockets was approximately 59% lower in these individuals compared to those with unhealthy lifestyle behaviors (4.2 ± 3.7% vs. 7.1 ± 3.7%). In line with these findings, a mean percentage of sites with PPD ≥ 6 mm of 4.4 ± 4.3% was observed following non-surgical therapy in a study involving systemically healthy patients with stage III/IV periodontitis and a healthy lifestyle [37]. A recent systematic review [39] indicated that non-surgical therapy typically achieves closure of about two-thirds of treated pockets (final PPD ≤ 4 mm), but sites with initial PPD ≥ 6 mm are less likely to reach this outcome due to the increased difficulty of effectively instrumenting the root surfaces as PPD increases [37, 40–43].

The differences in therapy outcomes based on lifestyle remained significant in the multiple logistic regression model, even after adjusting for baseline values (percentages of pockets with PPD ≥ 6 mm) and patient compliance (home biofilm control at 3 months), which could have influenced responses to treatment Steps 1 and 2 [44, 45]. In addition to similar mean FMPS values, clinical observations revealed no notable differences in plaque distribution patterns among lifestyle groups. These findings suggest a direct biological effect of lifestyle-related behaviors on periodontal health. Observational studies have established that modifiable lifestyle factors, including smoking, physical inactivity, obesity, and poor diet, are associated with the onset, severity, and progression of periodontitis [20, 46, 47]. Conversely, micronutrient intake and adherence to a Mediterranean diet have been shown to positively influence periodontal health [48–50]. However, the combined effects of these factors on periodontal health and therapy response remain poorly understood. As the literature suggests, it is essential to study lifestyle behaviors as interconnected factors, rather than in isolation. Unhealthy lifestyle habits (e.g., poor diet, smoking, high stress, and lack of physical activity) are linked to a higher risk of developing NCDs such as diabetes, cardiovascular diseases, and cancer, and may contribute to a shorter life expectancy [51, 52]. Regarding periodontal health, it has been observed that patients with periodontitis who engage in unhealthy lifestyle behaviors (unbalanced diet, low physical activity, high stress, poor sleep) derive less benefit from non-surgical treatment and are less likely to achieve therapy endpoints [23, 28, 53]. However, the majority of these studies included most individuals with moderate periodontitis (Stage II), whereas the inclusion of patients with severe periodontitis (stages III and IV) in the present cohort highlights the importance of lifestyle factors in this more susceptible population.

The underlying biological mechanisms connecting periodontitis and lifestyle likely involve inflammation and immune dysfunction. Shared molecular pathways can lead to LGSI, where the overproduction of ROS and pro-inflammatory mediators such as TNF-α and IL-6 negatively impact periodontal tissues, increasing susceptibility to the onset and progression of periodontitis [54]. Pro-inflammatory cytokines can cause systemic metabolic dysregulation. Notably, TNF-α inhibits insulin receptors, impairing the glucose uptake in peripheral tissues, while IL-6 contributes to insulin resistance by reducing adiponectin levels. These pathways, key in maintaining insulin sensitivity, are the bridge between periodontal inflammation and systemic conditions. Previous systematic reviews reported a reduction in the serum and gingival crevicular levels of these proinflammatory mediators after non-surgical instrumentation in patients with severe periodontitis and type 2 diabetes mellitus or obesity [55, 56]. Nonetheless, non-surgical therapy alone can alter the systemic inflammatory response triggered by periodontal pathogens, but it remains insufficient for complete resolution.

An imbalanced diet has been linked to a heightened inflammatory response in tissues. For instance, dietary fatty acids may modulate inflammatory response by altering both the structure and function of plasma membrane lipid rafts [14]. Intracellularly, fatty acids originating from the cell membrane can regulate inflammatory signaling by modulating NF-kb and PPARα/γ transcription factor pathways [57]. Furthermore, converging evidence from in vitro models, cross-sectional surveys, and randomized controlled trials underscores a pro-inflammatory role for saturated fatty acids (SFAs). Moreover, various papers highlighted the potential role of micronutrients (e.g. vitamins, calcium, zinc, magnesium, and other minerals) in periodontal pathogenesis [49, 58–62]. Diets with low adherence to the Mediterranean diet—characterized by the frequent consumption of pro-inflammatory foods such as processed meats and refined carbohydrates—promote weight gain and elevate ROS levels [63]. Notably, in our study, high BMI values, indicative of overweight, negatively influenced the response to periodontal therapy. Previous studies have also shown that higher BMI is linked to poorer responses to therapy, with more residual pockets with PPD ≥ 6 mm [64]. Excessive body fat accumulation leads to LGSI, which negatively affects both periodontal and systemic health [65].

Although the precise mechanisms linking physical activity to NCD – including periodontitis – remain to be fully elucidated, high levels of leisure-time physical activity have been consistently associated linked to reductions in both systemic and periodontal inflammatory responses [66–70]. Conversely, elevated occupational demands (OPA) may precipitate physical decline by chronically activating the hypothalamic–pituitary–adrenal axis, leading to sustained cortisol secretion, increased pro-inflammatory cytokine levels, and heightened oxidative stress [71]. Nevertheless, the net positive effect observed in the active group suggests that, in this cohort, the protective benefits conferred by higher levels of leisure-time physical activity likely predominate over any potential adverse effects associated with elevated occupational demands. This finding may indicate that voluntary, health-oriented physical activity exerts more favorable periodontal physiological adaptations, including improved vascularization, modulation of local inflammatory responses, and enhanced healing capacity [66].

The detrimental effects of smoking and alcohol on the periodontium are well documented. Smoking encourages the colonization of pathogenic periodontal bacteria and increases the expression of virulence factors. It also triggers the overproduction of ROS and cytokines, disrupting immune function [72]. Similarly, alcohol consumption alters immune responses, producing excessive cytokines and negatively affecting the periodontium [73]. Encouraging a healthy lifestyle could significantly reduce the burden of NCDs, including periodontitis, and improve treatment outcomes [28, 47]. This study supports the EFP/ORCA Consensus Report, which advocates integrating dietary education and personalized dietary regimens into the treatment plans of patients with oral inflammatory diseases and caries [74, 75]. However, evidence supporting the effectiveness of targeted lifestyle interventions during steps 1 and 2 of periodontal therapy remains limited [11].

Several limitations should be considered when interpreting these findings. First, lifestyle factors were assessed only at baseline, preventing the evaluation of any change over the 3-month study period. Second, the use of self-reported lifestyle data may introduce information bias, and the test-retest reliability of the questionnaire was not assessed, potentially affecting the consistency of the behavioral data. Third, the observational design of the study precludes causal inferences, and residual confounding may persist despite adjustment through multiple logistic regression. Additionally, relevant variables such as perceived stress and sleep quality, which may influence periodontal treatment outcomes, were not assessed. The study also exclusively enrolled cigarette smokers, limiting the generalizability of the findings to users of other tobacco products. Moreover, in the lifestyle scoring, both never-smokers and former smokers were categorized as “non-smokers”. While this categorization aligns with methodological approaches adopted in previous studies employing composite lifestyle scores [20, 26, 70], the possibility of residual risk among former smokers with shorter abstinence periods cannot entirely ruled out. Finally, the 3-month follow-up period primarily captures the early response to non-surgical periodontal therapy and may not reflect the long-term impact and sustainability of lifestyle-related clinical improvements. Previous studies have demonstrated that while significant clinical improvements often occur within the first 3 months post-therapy [42, 76, 77], longer-term follow-ups are necessary to assess the stability and maintenance of these improvements, especially following Steps 1 and 2 of therapy [45].

## CONCLUSIONS

This study suggests that a healthy lifestyle may contribute to better clinical outcomes following steps 1 and 2 of periodontal therapy in patients with severe periodontitis. The association between lifestyle and treatment outcomes appears independent of home hygiene levels, underscoring the importance of encouraging patients to quit smoking and adopt healthier dietary and physical activity habits. Further research, including multicenter prospective studies with larger sample sizes, is necessary to confirm these findings and improve the generalizability of the results. Randomized controlled trials are also needed to evaluate the benefits of dietary counseling, weight loss interventions, and increased physical activity within the context of periodontal therapy.

## Supplementary Information

Below is the link to the electronic supplementary material.

Supplementary Material 1

## Data Availability

All datasets generated during and/or analyzed during the current study are available from the corresponding author upon reasonable request.
