# Supplementary material for: Impact of lifestyle behaviors on clinical outcomes of steps 1 and 2 of periodontal therapy in stage iii-iv periodontitis: a cohort study
Source: Clin Oral Investig. 2025 Nov 24;29(12):584. doi: 10.1007/s00784-025-06668-9 (PMC12644107; doi:10.1007/s00784-025-06668-9)
Supplement: Supplementary file 1 — Supplementary Material 1 [file 784_2025_6668_MOESM1_ESM.docx]

**Supplementary Table 1.** Overall changes of periodontal clinical variables over the 3 months of the study (mean ± standard deviation).

| **Variables** | **Baseline (T0)** | **3 months (T1)** | **Difference T0-T1** | **P-value** |
| --- | --- | --- | --- | --- |
| N° teeth | 26.0 ± 3.9 | 24.9 ± 4.4 | 1.1 ± 1.7 | 0.001 |
| FMPS (%) | 75.5 ± 17.1 | 14.4 ± 6.7 | 61.1 ± 17.6 | < 0.001 |
| FMBS (%) | 73.2 ± 20.0 | 15.7 ± 7.2 | 57.5 ± 19.9 | < 0.001 |
| PPD (mm) | 4.3 ± 0.7 | 3.0 ± 0.6 | 1.3 ± 0.7 | < 0.001 |
| % PPD ≥ 4 mm | 25.2 ± 11.3 | 11.4 ± 7.3 | 13.8 ± 11.5 | < 0.001 |
| % PPD ≥ 6 mm | 13.4 ± 6.7 | 5.7 ± 4.0 | 7.7 ± 6.6 | < 0.001 |
| REC (mm) | 0.7 ± 0.7 | 0.8 ± 0.7 | –0.1 ± 0.3 | 0.01 |
| CAL (mm) | 5.0 ± 1.0 | 3.8 ± 1.0 | 1.2 ± 0.8 | < 0.001 |

FMPS, Full Mouth Plaque Score; FMBS, Full Mouth Bleeding Score; PPD, Probing Pocket Depth; %PPD ≥ 4 mm, percentage of sites with probing depth of 4-5 mm and bleeding; % PPD ≥ 6 mm, percentage of sites with probing depth ≥ 6 mm; REC, Gingival Recession; CAL: Clinical attachment level.

**Supplementary Table 2.** Changes in periodontal variables from baseline to 3 months after therapy by lifestyle group (mean ± SD)

| **Variables** | **Lifestyle groups** | **Difference from baseline to 3 months** | **P*** | |
| --- | --- | --- | --- | --- |
| FMPS (%) | Unhealthy (score 0-1) | 60.4 ± 19.4 | 0.450 | |
|  | Moderately healthy (score 2) | 64.2 ± 15.8 |  |  |
|  | Healthy (score 3-4) | 59.6 ± 17.4 |  |  |
|  |  |  |  | |
| FMBS (%) | Unhealthy (score 0-1) | 53.8 ± 21.4 | 0.038^C^ | |
|  | Moderately healthy (score 2) | 53.9 ± 19.2 |  |  |
|  | Healthy (score 3-4) | 63.0 ± 16.3 |  |  |
|  |  |  |  |  |
| PPD (mm) | Unhealthy (score 0-1) | 1.1 ± 0.8 | 0.002^B^ |  |
|  | Moderately healthy (score 2) | 1.1 ± 0.6 |  |  |
|  | Healthy (score 3-4) | 1.6 ± 0.7 |  |  |
|  |  |  |  |  |
| % PPD ≥ 4 mm | Unhealthy (score 0-1) | 11.2 ± 11.1 | 0.040^C^ |  |
|  | Moderately healthy (score 2) | 11.9 ± 10.9 |  |  |
|  | Healthy (score 3-4) | 16.9 ± 11.7 |  |  |
|  |  |  |  |  |
| % PPD ≥ 6 mm | Unhealthy (score 0-1) | 7.7 ± 8.0 | 0.853^C^ |  |
|  | Moderately healthy (score 2) | 7.3 ± 6.1 |  |  |
|  | Healthy (score 3-4) | 8.0 ± 5.9 |  |  |
|  |  |  |  |  |
| REC (mm) | Unhealthy (score 0-1) | 0.2 ± 0.3 | 0.510 |  |
|  | Moderately healthy (score 2) | 0.1 ± 0.4 |  |  |
|  | Healthy (score 3-4) | 0.1 ± 0.5 |  |  |
|  |  |  |  |  |
| CAL (mm) | Unhealthy (score 0-1) | 0.9 ± 0.6 | 0.002^A^ |  |
|  | Moderately healthy (score 2) | 1.1 ± 0.7 |  |  |
|  | Healthy (score 3-4) | 1.5 ± 0.8 |  |  |

SD, Standard deviation; FMPS, Full Mouth Plaque Score; FMBS, Full Mouth Bleeding Score; PPD, Probing Pocket Depth; % PPD ≥ 4 mm, percentage of sites with probing depth of 4-5 mm and bleeding; % PPD ≥ 6 mm, percentage of sites with probing depth ≥ 6 mm; REC, Gingival Recession; CAL: Clinical attachment level; *P value for comparisons among groups; Superscripts indicate statistically significant differences between healthy and unhealthy groups: ^A^ < 0.001; ^B^ < 0.01; ^C^ < 0.05.

**Supplementary Table 3.** Sensitivity analysis for predictors associated with primary treatment outcome (a maximum of 4 sites with PPD ≥ 5 mm at T1).

| **Variables** | **OR** | **CI 95%** | **P-value** |
| --- | --- | --- | --- |
| Age (years) | 0.98 | 0.95 - 1.02 | 0.290 |
| Gender |  |  |  |
| Female | Ref. |  |  |
| Male | 1.39 | 0.61 – 3.16 | 0.431 |
| Hypertension |  |  |  |
| No | Ref. |  |  |
| Yes | 0.67 | 0.22 – 1.99 | 0.472 |
| FMPS at T1 (%) | 1.01 | 0.95 – 1.08 | 0.682 |
| % PPD ≥ 6 mm at T0 | 0.90 | 0.84 – 0.97 | 0.005 |
| Lifestyle patterns |  |  |  |
| Unhealthy | Ref. | - | - |
| Moderately healthy | 1.19 | 0.38 – 3.75 | 0.770 |
| Healthy | 3.35 | 1.18 – 9.47 | 0.023 |
| BMI (kg/m^2^) |  |  |  |
| Overweight | Ref. | - | - |
| Normal weight | 2.82 | 1.03 – 7.19 | 0.044 |
| Intercept | 3.09 | - | 0.392 |

PPD, Periodontal pocket depth; FMPS, Full Mouth Plaque Score; T0, baseline; T1, 3 months after Steps 1 and 2 of periodontal therapy; % PPD ≥ 6 mm, percentage of sites with probing depth ≥ 6 mm; BMI, Body mass index; OR, Odds ratio; 95% IC, 95% Interval confidence.
